# Digital design of functional surgery for odontogenic cyst intruding into maxillary sinus^☆^

**DOI:** 10.1016/j.bjorl.2017.02.003

**Published:** 2017-03-19

**Authors:** Ying Kai Hu, Chi Yang, Guang Zhou Xu, Qian Yang Xie

**Affiliations:** Ninth People's Hospital, Shanghai Jiao Tong University, School of Medicine, Department of Oral and Maxillofacial Surgery, Shanghai, China

**Keywords:** Maxillary sinus, Sinus membrane, Bone plate, Surgical flaps, Seio maxilar, Membrana sinusal, Placa óssea, Retalhos cirúrgicos

## Abstract

**Introduction:**

Traditional Caldwell-Luc approach needs modifications for odontogenic cysts intruding into the maxillary sinus, to preserve sinus mucosa and bony contour. Recently, digital technology has been widely applied to the field of maxillofacial surgery, guiding the surgical plan and improving its accuracy.

**Objective:**

This study attempted to present and evaluate the functional surgery of odontogenic cysts intruding into the maxillary sinus using a computer-assisted pre-surgical design.

**Methods:**

Consecutive patients with odontogenic cysts intruding into the posterior part of the maxillary sinus were enrolled. Method I “Bony wall reimplantation method” was performed for large lesions exceeding the zygomatic alveolar crest but without apparent bone destruction of the anterior wall of the sinus, while Method II “bone removal method” was more convenient for small lesions near to the zygomatic alveolar crest. The gap was filled with a pedicled buccal fat pad after lesion removal and all cases were without inferior meatal antrostomy.

**Results:**

A total of 45 cases were included in the study. 22 were operated using method I while 23 were operated with method II. Operations were completed in 20 min. Pain disappeared in 3.62 days on average, and swelling 6.47 days. Nasal bleeding occurred in 8 patients lasting 1–3 days. Suppurative inflammation was observed in 1 patient, and infection occurred after bone reposition. Other repositioned free bony wall was without resorption in CT images.

**Conclusions:**

Sinus mucosa and bony wall should be conserved. Preoperative digital design can guide osteotomy effectively during the surgery. Bone reposition is not suitable for suppurative inflammation. The pedicled buccal fat pad is enough for drainage and inferior meatal antrostomy is not necessary.

## Resumo

**Introdução:**

A abordagem tradicional de Caldwell-Luc precisa de modificações para os cistos odontogênicos que se introduzem no seio maxilar, para preservar a mucosa sinusal e o contorno ósseo. Recentemente, a tecnologia digital tem sido amplamente aplicada ao campo da cirurgia maxilofacial, orientando o plano cirúrgico e melhorando sua precisão.

**Objetivo:**

Esse estudo teve como objetivo apresentar e avaliar a cirurgia funcional de cistos odontogênicos intrusivos no seio maxilar utilizando um desenho pré-cirúrgico assistido por computador.

**Método:**

Foram recrutados pacientes consecutivos com cistos odontogênicos intrusivos na parte posterior do seio maxilar. O Método I, “método de reimplante de parede óssea”, foi realizado em lesões grandes que excediam a crista zigomático-alveolar, mas sem destruição óssea aparente da parede anterior do seio, enquanto o método II, “método de remoção óssea” foi mais conveniente para pequenas lesões próximas à crista zigomático-alveolar. O espaço foi preenchido com um retalho pediculado do corpo adiposo bucal após a remoção da lesão e todos os casos foram realizados sem antrostomia meatal inferior.

**Resultados:**

Um total de 45 casos foram incluídos no estudo. Vinte e dois foram submetidos à cirurgia utilizando-se o método I, enquanto que 23 foram submetidos ao método II. As operações foram concluídas em 20 minutos. A dor desapareceu em média após 3,62 dias, e o edema, depois de 6,47 dias. Hemorragia nasal ocorreu em 8 pacientes com duração de 1 a 3 dias. Processo supurativa foi observado em 1 paciente ocorrendo após a reposição óssea. Outros retalhos reposicionados livres da parede óssea não mostraram reabsorção em imagens de TC.

**Conclusões:**

A mucosa sinusal e a parede óssea devem ser preservadas; o desenho digital pré-operatório pode orientar a osteotomia de forma eficaz durante a cirurgia; a reposição óssea não é adequada em processos supurativos. O retalho pediculado de corpo adiposo bucal é suficiente para a drenagem e antrostomia meatal inferior não é necessária.

## Introduction

Maxillary sinus is vulnerable to be invaded by odontogenic cystic lesions owing to the anatomical relation to the upper alveolar bone.^1^, ^2^ These lesions usually intrude into the sinus through the inferior and posterior walls. Management of the maxillary sinus diseases is generally via Caldwell-Luc operation or functional endoscopic surgery.

However, cysts that are close to the osteomeatal complex can be removed endoscopically, while those lesions lying laterally or posteriorly are more easily removed by Caldwell-Luc approach, which provides a direct visualization and convenient manipulation. Medial and anterior parts of the sinus and the alveolar recess are difficult to access endoscopically, therefore, a complete enucleation may not be assured through an endonasal approach alone.^3^ In addition, odontogenic cysts often require teeth extraction which may be performed only through an oral approach.^2^ Consequently, Caldwell-Luc operation is superior to the endoscopic procedure in cases of odontogenic cysts intruding into the anterolateral or posterior part of the maxillary sinus.

The standard Caldwell-Luc procedure facilitates an access to the maxillary sinus through the canine fossa, which provides an optimal visualization of the anterior or inferior sinus walls, but for the posterolateral wall, it is a little difficult to operate under direct view, leaving some remnants of the cyst wall and a high recurrence rate. Furthermore, a radical removal of the sinus membrane and a permanent bone defect at the anterior sinus wall might cause a considerable blood loss and prolonged operation time during the surgery, meanwhile, higher complication rates including lingering pain and swelling, facial or dental paraesthesia, facial deformity and chronic maxillary sinusitis.^4^ Several modifications have been reported in literatures, including bony wall reimplantation, sinus membrane preservation or without an inferior meatal antrostomy.^4^, ^5^

Recently, digital technology has been widely applied to the field of maxillofacial surgery, guiding the surgical plan and improving its accuracy.^6^, ^7^ The purpose of the current study was to refine our experience and address our philosophy on the conservative treatment of cystic lesions intruding into the posterior part of the maxillary sinus with computer-assisted techniques, and to assess the intraoperative effectiveness and postoperative outcomes.

## Methods

### Study design

All procedures performed in studies were in accordance with the ethical standards of our hospital (N° 2016-57-T14) and with the 1964 Helsinki declaration and its later amendments or comparable ethical standards. The study population included patients presenting with odontogenic cystic lesions intruding into the posterior part of the maxillary sinus from January 2012 to December 2015, who underwent a functional surgery performed by the same surgeon, with their surgical plans made digitally. Patients were excluded as study subjects if: (1) the lesion was diagnosed as a malignant tumor; (2) they have had previous nasosinusal procedures; (3) the lesion was near the ostium-meatal complex, which were easier removed endoscopically; (4) the lesion was huge and the boundary between it and sinus mucosa was ill-defined, which made it difficult to preserve sinus mucosa. Informed consent was obtained from all individual participants included in the study.

### Digital techniques

#### Data capture

**Figure 1:**
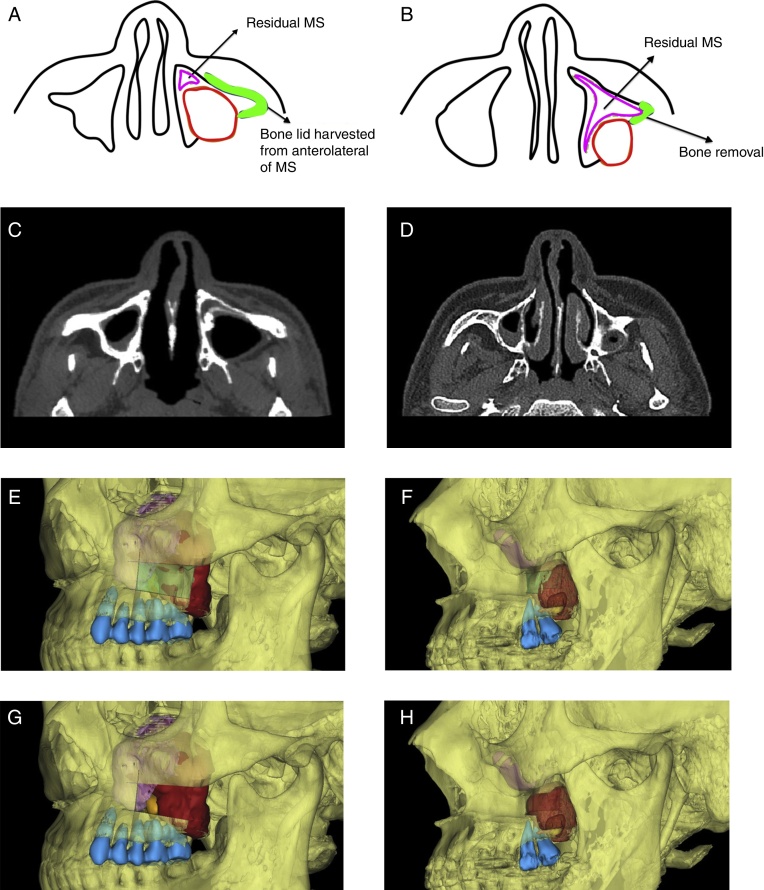
Diagram of surgical approach. A, C, E and G, For a large lesion, anterolateral bony wall of maxillary needs to be removed to distinguish boundary of sinus mucosa and cyst wall, and to remove the lesion; B, D, F and H, For a relatively small lesion, only a small amount of bone posterior to zygomaticoalveolar crest removal is enough (the red area shows the cyst, purple area shows affected sinus, orange area shows tooth in the cyst, green area shows the bone plate removal, and blue area shows maxillary teeth of the affected side). MS, maxillary sinus.

The patients’ preoperative Computed Tomography (CT) scans were stored (layer thickness, 0.625 mm) as Digital Imaging and Communications in Medicine on a disk, then imported into the computer to perform the 3D reconstruction using ProPlan CMF, version 1.3 (Materialize Medical, Leuven, Belgium) software. With the assistance of the preoperative digital design, the residual affected maxillary sinus, cystic lesion and ipsilateral maxillary teeth were easily separated and marked with different colors (Fig. 1).

#### Osteotomy design

The osteotomy lines were designed according to the measurements of the sinus and cystic lesion. For a large lesion extending over the zygomaticoalveolar crest with no obvious bone destruction of the anterolateral wall of the sinus, rectangular osteotomy lines were designed on the anterolateral wall (Fig. 1). Because of the large volume of the posterior part of the lesion, the medial cut could be shorter than the distal cut, and the upper and lower cuts were inclined upward and downward. Meanwhile, it would be better for the medial cut to expose the boundaries of the cyst wall and sinus mucosa, and the upper cut should not be too high to protect the infraorbital nerve and leave a space for the titanium miniplate fixation, also the lower cut was made with a safety margin of at least 2 mm from the root tips of the upper teeth.

For a relatively small lesions mostly located distal to the zygomaticoalveolar crest, removal of small amount of the bone distal to the crest was enough for lesion exposure (Fig. 1).

### Surgical approach

Following the injection of 2% lidocaine with 1:100,000 epinephrine into the pterygopalatine canal and sublabial area, an access to the maxillary sinus was started at the mucogingival border line via an incision of the mucosa down to the bone. Selection of the operation type was done on the basis of the pre-surgical computer-assisted design.

#### Bony wall reimplantation method

**Figure 2:**
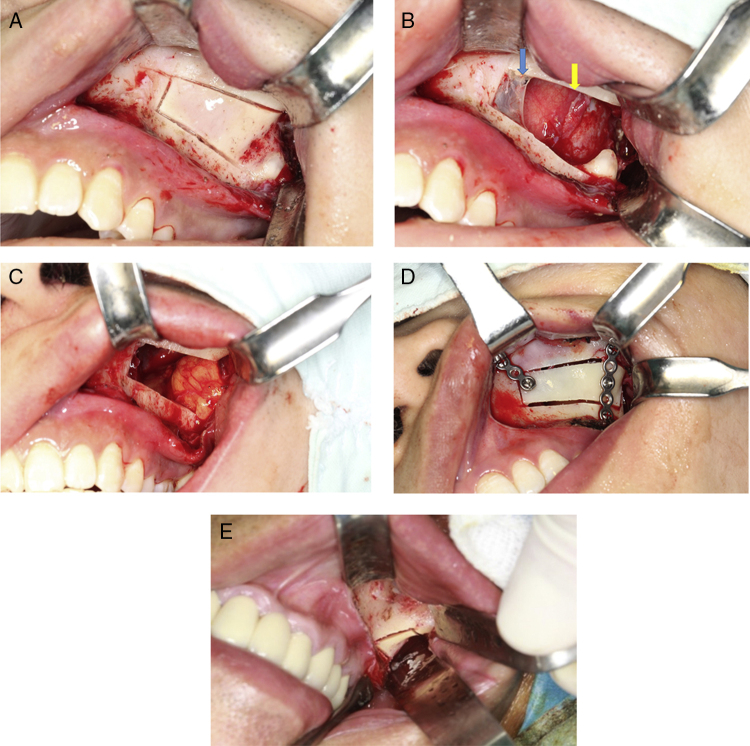
Surgical approach. (A) Bony wall reimplantation method: use piezosurgery to make a window anterolateral wall of the maxillary sinus, and cut medial, upper and lower osteotomy line first. (B) Lesion exposure after bone removal (the blue arrow shows sinus mucosa, and yellow arrow shows cyst wall). (C) Fill pedicled buccal fat pad flap into bone cavity. (D) Bone plate was repositioned and fixed by mini titanium plates. (E) Bone removal method: make a window lateral to zygomaticoalveolar crest.

When the cystic lesion was large, a bony window was made on the anterolateral wall of the maxillary sinus by piezosurgery. Medial, upper and lower cuts were executed first only in the cortical bone, avoiding damage of the sinus mucosa (Fig. 2). Before lifting the bone lid to expose the boundary of the sinus mucosa and cyst wall and putting it in a saline solution, a titanium miniplate was adapted to the bone lid to facilitate subsequent reposition (Fig. 2). The window created by this procedure allowed an excellent visibility of all anatomical details and a wide access for manipulation. The cystic lesion was easily removed and sinus mucosa was left in place. Afterwards, the sinus was irrigated with a physiologic solution and a pedicled buccal fat pad (BFP) was harvested and filled into the bone cavity. The bone lid was finally reimplanted and fixed with the titanium miniplate. When there was an excessive exudation, iodoform gauze was placed for drainage. Once the lid had been fixed in its original position, the covering periosteum and soft tissues were likewise returned to the previous position sutured by 4–0 absorbable vicryl.

#### Bone removal method

When the cystic lesion was relatively small, a small amount of bone of the posterior wall of the maxillary sinus and distal to the zygomaticoalveolar crest was removed with piezosurgery or rongeur forceps. After clearing the lesion, there was no need to reposition the removed bone, only filling the gap with a pedicled BFP would be enough.

### Intraoperative assessment

The intraoperative assessment included the followings: (1) anesthetic effect and operative blood loss, (2) whether the lesion could be removed smoothly and preserve the sinus mucosa at the same time, (3) if the bone lid could be repositioned adequately, (4) operation time, (5) others such as if there was suppurative infection or excessive exudation and so on.

### Postoperative evaluation

Postoperative assessment included duration of pain, swelling, nasal bleeding and infection. CT scan was taken to observe the condition of the bone lid and maxillary sinusitis 3 months after the operation.

## Results

Totally 45 patients were included in the study, their ages ranged from 17 to 68 years (mean, 43.26 years). There were 27 males and 18 females. In 22 patients, bony wall reimplantation method was performed, while the bone removal method was used in the other patients.

All surgeries were completed in 20 min, and the intraoperative anesthetic effect was perfect without abortion of the surgical procedure because of pain. The amount of intraoperative blood loss was small except in 2 cases manifested with an impulsive bleeding due to damage of the posterior superior alveolar artery during cyst wall scraping. All lesions were removed completely with sinus mucosa preservation in accordance with the pre-surgical computer-assisted plan. The bone lids were repositioned smoothly in all 22 cases, with the use of iodoform gauze because of excessive exudation in 2 cases, and also suppurative infection was seen in 1 case of which the bone surface was rough and oozing blood.

**Figure 3:**
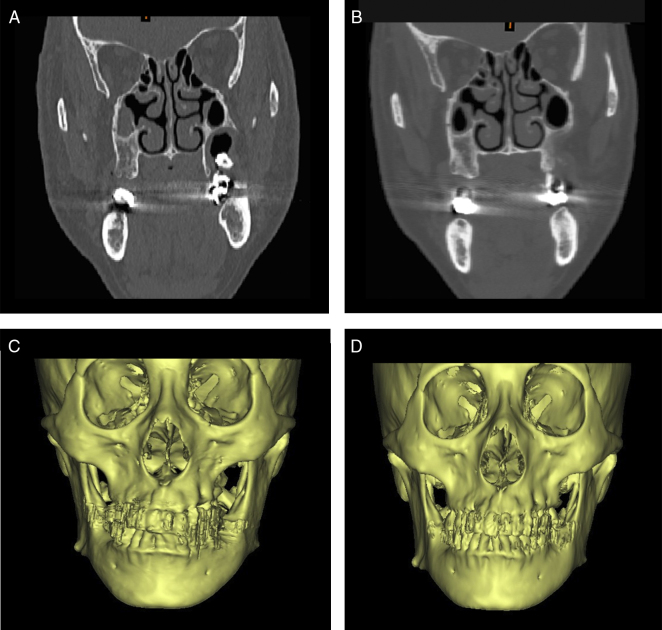
CT and 3D reconstruction images of bone removal method. (A and C) Before operation. (B and D) Images postoperatively shows normal shape.

**Figure 4:**
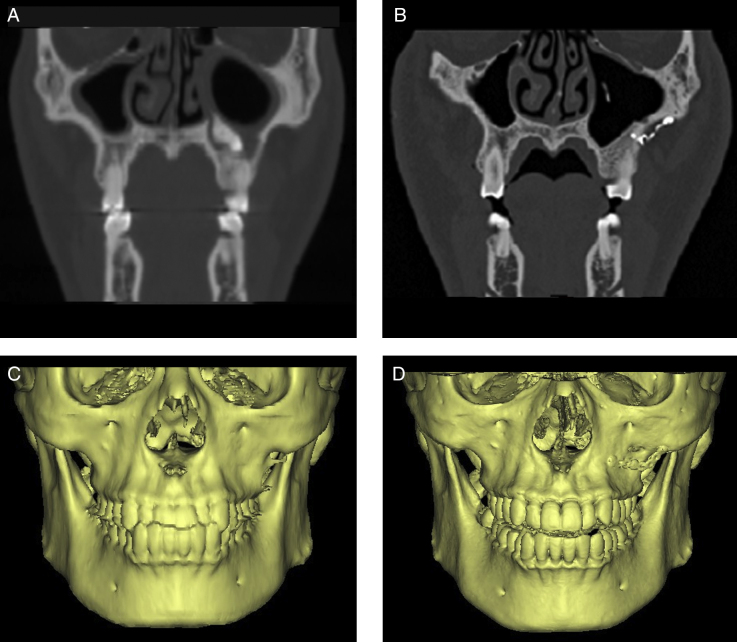
CT and 3D reconstruction images of bony wall reimplantation method. (A and C) Before operation. (B and D) Images post-operatively showing normal shape and no bone resorption.

The durations of pain and swelling (all cases without infraorbital involvement) were 2–7 days (mean 3.62 days) and 5–14 days (mean 6.47 days) respectively. There were 8 cases presenting with nasal bleeding for 1–3 days. Postoperative infection occurred in one case that had had suppurative infection and the clinical symptoms disappeared after removing the titanium miniplate and bone lid and draining for 3 months. Follow-up CT scans showed no obvious resorption of other bone lids and no obvious change of maxillary contour (Figure 3, Figure 4), and only 2 cases presented with mild sinus mucosal thickening.

## Discussion

Classic Caldwell-Luc surgery is characterized by three principal features: access via the lateral wall of the maxillary sinus, surgical removal of the sinus mucosa, and establishment of a drainage channel into the lower nasal cavity.^8^ Criticism of this procedure has focused on the radical removal of the sinus mucosa and a remaining permanent bone defect, leading to more blood loss and operation time as well as higher complication rates including facial swelling, cheek discomfort, fever, facial asymmetry, facial paresthesia and recurrent sinusitis.^9^, ^10^ Accordingly, in order to treat odontogenic sinus pathologies, we performed a functional surgery with the intention of retaining the sinus mucosa and bony structures; so that the normal physiologic function and contour can be preserved and the postoperative reactions can be reduced.

The maxillary nerve exits the middle cranial fossa through the foramen rotundum to enter the pterygopalatine fossa where it gives off several branches to the maxillary teeth and maxillary sinus.^11^ So injecting anesthetic into the pterygopalatine canal, adding local anesthesia near the maxillary tuberosity and labial area could acquire an excellent anesthetic effect, which made it possible to perform the surgery under local anesthesia instead of general anesthesia, which was proved by our study. None of the surgeries were aborted because of poor anesthetic effects. However, pterygopalatine canal injection seemed to be difficult for less experienced operators.

In recent years, the computer-assisted design technique has been widely used in the pre-surgical design of oral and maxillofacial surgeries, with the advantages of simplicity, high precision and time saving.^12^ With the help of ProPlan CMF software (Materialize Medical, Leuven, Belgium), we could not only observe the location and size of the cystic lesions as well as the surrounding significant anatomic structures visually, but also the osteotomy could be simulated to guide the surgery, contributing to less invasive, more accurate and simpler surgery, especially for the inexperienced physicians. Our study showed a perfect protection of the sinus mucosa, bony structure and root tips of the maxillary teeth.

Classical Caldwell-Luc, in which the antral lining has been completely removed, could be detrimental to the sinus physiology because the mucociliary lining is replaced with a nonfunctional mucosa, with changes of the bony structures.^2^, ^13^ There has also been an agreement that the sinus membrane will recover once a proper ventilation is restored.^14^ Consequently, preserving the sinus mucosa in place is very important, and together with the computer-assisted pre-surgical design, it can lead to less operation time, less blood loss and less trauma.

To obtain clearance of the lesion and preserve the sinus mucosa simultaneously, it is essential to identify the boundary of both. When the volume of the lesion invading the posterior wall of the maxillary sinus is small, removal of a small amount of bone distal to the zygomaticoalveolar crest is just enough, and it will not cause an obvious malformation (Fig. 3). While for big lesions extending over the crest, it seems impossible to distinguish the cyst wall and the sinus mucosa directly without removing the anterolateral wall of the maxillary sinus. Therefore, a bone window in the anterolateral maxillary wall was performed to obtain a better visualization. If the defect resulting from this surgical intervention remains open, the volume of the maxillary sinus will be reduced by the inward collapse of the soft tissues of the cheek. Moreover, the scar contraction of the tissues near the site of entry of the infraorbital nerve may cause irritation leading to neuralgic pain.^5^

Immediate reclosure of the defect is the most important and effective method to prevent the intrusion of the soft tissue into the maxillary sinus. It is obviously better to use the bone piece obtained during creation of the surgical access to the maxillary sinus, the shape of which lends itself most naturally to reclosure of the defect. This has been achieved by Abello creating a cranially pedicled flap of periosteum and bone, obtained by making three consecutive bur-cuts in the shape of the letter “U” and fracturing of the fourth side of the rectangle subsequently, resulting in a flap hinged on the periosteum. Disadvantages of this procedure are the impossibility of the exact prediction of the line of fracture and the danger of the fracture extending into the infraorbital foramen. Moreover, the hinged flap forms an additional obstruction in the surgical field. Lindorf has reported the removal of the bone piece during the surgical access by inclining the cuts at an angle in order to obtain a specimen immediately reusable as a free implant for defect closure, and the bone lid was secured by suturing using an absorbable catgut or by means of an adhesive based on fibrin.^15^ However, the downsides to this approach were complex operation, high precision of cut angles, and worse retention for large bone piece. In our method in the current study, we performed the osteotomy using the piezosurgery, which offered the advantages of handy operation, precise cutting, soft tissue and nerve protection, less heat production, and less bone loss. Using titanium miniplates which possessed good biocompatibility for fixation could achieve an excellent retention and simplify the procedure. In addition, the follow-up radiological evaluation demonstrated no pronounced malformation and bone resorption (Fig. 4).

Owing to the rich blood supply, the pedicled BFP possesses a strong anti-infectious ability. Other advantages are the versatility, low rate of complications, minimal damage to the donor site morbidity, inhibiting scar formation, and quick surgical technique because it is located in the same surgical field as the cavity to be filled. In addition, the quick epithelialisation of the uncovered fat is a characteristic feature of the pedicled BFP flap and is histologically proven,^16^, ^17^ therefore it can facilitate the physiological and functional recovery of the maxillary sinus. Despite lacking of a control study, our study still can indicate that filling the bone cavity with a pedicled BFP, adding iodoform gauze if needed, is sufficient for drainage, and there is no need to perform the inferior meatal antrostomy.

With the application of the computer-assisted pre-surgical plan, the functional surgery for the odontogenic cysts intruding into the posterior part of the maxillary sinus could provide an easier manipulation, excellent preservation of the sinus mucosa and bone structure, as well as fewer postoperative reactions. Further studies on the long-term evaluation and quantitative measurement of the volume changes of the maxillary sinus will be summarized in our next study.

## Conclusion

The functional surgery is effective and inferior meatal antrostomy is not necessary. The pedicled BFP is sufficient for drainage and would not influence sinus cavity. Preoperative digital design can guide osteotomy effectively during the surgery. Bone reposition is not suitable for suppurative inflammation.

## Conflicts of interest

The authors declare no conflicts of interest.
